# Easier Access to Acute Care and Lower Stroke Mortality: What is Next?

**DOI:** 10.31662/jmaj.2025-0068

**Published:** 2025-03-21

**Authors:** Hideo Yasunaga

**Affiliations:** 1 Department of Clinical Epidemiology and Health Economics, School of Public Health, The University of Tokyo, Tokyo, Japan

**Keywords:** stroke, acute care density index, time from onset to hospital arrival

Patients living in areas with limited access to short-term care may not receive prompt treatment. However, the association between access to short-term care and stroke outcomes remains controversial.

A study by Sasahara et al.^(1)^, published in the *JMA Journal*, aimed to determine the association of acute care density index (ACDI) and home-to-hospital distance with in-hospital mortality in patients with acute stroke. I believe the descriptive statistics in their study may provide new knowledge on the status of short-term care for stroke. Very low ACDI scattered in rural areas reflects an absolute shortage of medical resources. Low ACDI is concentrated in metropolitan areas, reflecting relative shortages due to high population density and demand for medical care. In some rural areas, ACDI is high, possibly because of a surplus of resources relative to population size, suggesting the need for redistribution. Long distance from home to hospital was observed mainly in less populated areas. In contrast, distances were shorter in metropolitan areas, highlighting the proximity to hospitals.

Multivariable regression analyses showed lower ACDI was associated with higher in-hospital mortality in urban and rural areas, but not in depopulated areas. Those analyses also showed somewhat unexpected results that home-to-hospital distance was not associated with in-hospital mortality in urban and rural areas, and home-to-hospital distance exceeding the median was rather associated with lower in-hospital mortality in depopulated areas.

In our view, the multivariable analyses for ACDI in urban and rural areas provide a novel finding; however, any other multivariable analyses may have little meaning. Interpretation of the results should be that ACDI may be a good predictor of stroke outcomes only in urban and rural areas but not in depopulated areas. Home-to-hospital distance is not useful for predicting stroke outcomes.

The authors explain a reason for the unexpected association of ACDI and home-to-hospital distance with stroke outcomes may be the well-developed emergency systems and the use of doctor helicopters for long-distance transport in depopulated areas. I agree with their comment; moreover, in the first place, these factors were not measured in their study, and the results were biased by these unmeasured confounders. Quality of emergency systems and their related factors (e.g., dispatch and triage systems, shortage of medical resources, insufficient infrastructure) can affect outcomes, but their study did not adjust for any such factors. Moreover, a serious question is raised about whether home-to-hospital distance is a valid indicator for predicting the outcomes of stroke. “Time from onset to hospital arrival” can be the best predictor of receiving reperfusion therapy and mortality ^(2)^. The results suggest that home-to-hospital distance may not necessarily be associated with time from onset to hospital arrival, and assessing home-to-hospital distance has little meaning, particularly for depopulated areas. Future studies should seek to collect data on time from onset to hospital arrival to investigate the relation between area-related factors and outcomes of stroke.

I acknowledge that the associations between lower ACDI and higher mortality in urban and rural areas were “statistically significant.” However, the authors did not discuss whether the associations were “clinically important” or had any health policy implication. Crude mortality in the ACDI Q1 and Q4 was 4.03% vs 5.40% in urban areas and 5.08% vs 6.32% in rural areas, respectively. Odds ratio (95% confidence interval) for ACDI Q4 with reference to Q1 was 1.354 (1.193-1.538) in urban areas and 1.259 (1.112-1.425) in rural areas.

The authors should carefully discuss whether only an approximately 1% difference in mortality between the areas with the lowest and highest ACDI is clinically important. They should also consider whether 1% difference is acceptable for residents in the lowest ACDI areas and discuss the costs required for improving ACDI to achieve 0% difference in mortality, and the costs acceptable to tax payers.

## Article Information

### Conflicts of Interest

None
